# Wild Ungulate Prey Size and Feeding Group Demographic Structure Affect Interactions and Per Capita Food Intake of African Pride Lions in East African Maasai Steppe

**DOI:** 10.1002/ece3.71787

**Published:** 2025-07-28

**Authors:** Thobias Oddo Tomeka, Bernard M. Kissui, Ifura Godfrey Ukio, Frank R. Mushi, Rudolf F. Mremi, Nathan J. Roberts, Marcel Holyoak, Guangshun Jiang

**Affiliations:** ^1^ Feline Research Center of National Forestry and Grassland Administration, College of Wildlife and Protected Area Northeast Forestry University Harbin China; ^2^ College of African Wildlife Management Mweka Moshi Tanzania; ^3^ The School for Field Studies Center for Wildlife Management Studies Karatu Tanzania; ^4^ Maasai Steppe Carnivore Conservation Trust Arusha Tanzania; ^5^ Department of Geography Kings College London London UK; ^6^ Northeast Asia Biodiversity Research Center Northeast Forestry University Harbin China; ^7^ Department of Environmental Science and Policy University of California Davis California USA

**Keywords:** African lion, carcass biomass, demographic structure, feeding group, per capita food intake

## Abstract

The African lion ( *Panthera leo* Linnaeus, 1758) has evolved complex associations in which individual survival is an interplay of cooperation among pride members. Although feeding behaviors of African lions are widely known, our understanding of how age and sex classes affect per capita intake of other individual members in a pride remains unclear. This study used long‐term population monitoring data collected from 2004 to 2023 to assess how prey size and feeding group composition affect different age/sex class interactions and per capita food intake of African lion cubs, subadults, adult males, and adult females. The results indicate that African lion feeding group composition and interaction patterns at carcasses were affected by prey size. Cub per capita food intake was reduced by increased numbers of cubs and subadults. For subadults, the per capita intake was reduced as the number of cubs, subadults, and adult females increased. However, subadults increased their per capita intake rates when feeding together with both cubs and adult females. Adult females also showed competitive interactions when feeding at carcasses with cubs, subadults, and fellow adult females. Nevertheless, the adult female per capita intake was increased when more females fed with cubs or subadults and when feeding in combination with cubs and adult males. For adult male lions, only increased numbers of adult females led to a reduced per capita intake at carcasses, reflecting competition. These varied effects on per capita food intake suggest how age and sex‐based composition of feeding groups play a role in the foraging success of African lions and how food availability may influence the demographic composition of prides.

## Graphical abstract

This study used long‐term lion population monitoring data in the Tarangire–Manyara ecosystem to understand how feeding group demographic structure affects per capita food intake for cubs, subadults, adult females, and adult males. Our results revealed that the composition of feeding groups in carcasses variably affects per capita food intake for each demographic group, with both competition as well as facilitation interactions in certain age/sex class individuals. These findings emphasize the necessity of maintaining prey populations as a means of ensuring conservation of threatened African lions.

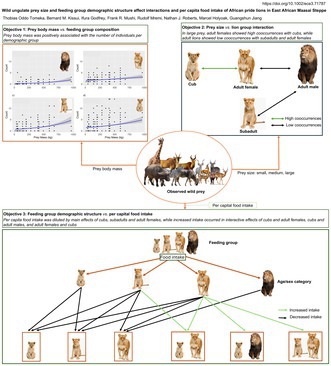

## 1 Introduction

In many mammals, foraging success is related to fitness (Beltran et al. 2023; Jeanniard‐Du‐dot et al. 2017; Oksanen and Lundberg 1995; Ritchie 1990). The behavior and energy intake of a forager are directly linked to its fitness with an impact on its contribution to the next generation (MacArthur and Pianka 1966; Pyke 1984). However, it is argued that life history aspects should be considered when assessing an animal's foraging adaptations (Morse and Fritz 1987). Most predators are opportunistic hunters (Elbroch and Wittmer 2013) and play important roles in regulating the numbers of their herbivore prey, which reduces grazing pressures on ecosystems. Despite this important role, most ecosystems have been altered in ways that disrupt prey community structure and populations of flagship predators such as the Amur tiger (Jiang et al. 2014; Li et al. 2017; Long et al. 2021; Tian et al. 2014), snow leopard (Forrest et al. 2012; Ghoshal et al. 2019; Hussain 2003; Li et al. 2013; Li and Lu 2014; Mahmood et al. 2019), and the African lion (*Panthera leo*) (Bauer et al. 2022; Coals et al. 2020; Everatt et al. 2019; Henschel et al. 2014; Kissui and Packer 2004; Lindsey et al. 2017; Loveridge et al. 2022; Nicholson, Bauer, et al. 2023; Nicholson, Dickman, et al. 2023; Smitz et al. 2018).

**FIGURE 1:**
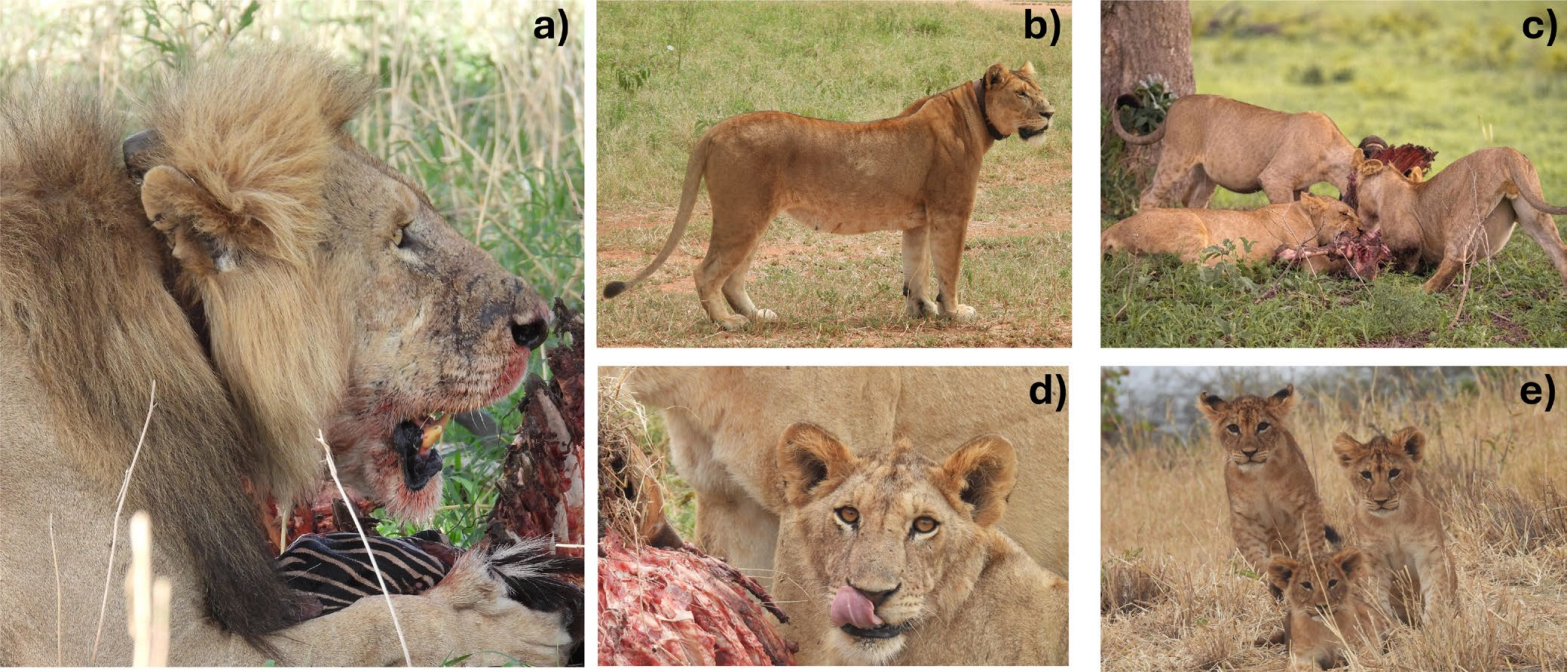
African lion ( *Panthera leo* ) in the Tarangire–Manyara ecosystem; (a) A collared adult male feeding on zebra carcass; (b) a collared adult female; (c, d) subadult lions feeding on a carcass; and (e) cubs. Photographs taken during surveys of the long‐term population monitoring program of the African lion in the Tarangire–Manyara ecosystem. Photo credits: Frank R. Mushi and George Shango.

African lions shown in Figure 1 (focal species for this study) are considered highly sociable animals, with group living reported to influence group and individual fitness (Kissui et al. 2010; Packer 2019; Palmer et al. 2023). However, their organization is flexible with evidence of occasional solitary breeding in some populations (Bygott et al. 1979; Packer 1986). Group living is adaptative in securing territories, accessing mates, rearing cubs, and in cooperative hunting (Packer and Pusey 1997). Male lions benefit from cooperation by providing competitive advantage through retaining tenure in female prides, mating with more females and producing more surviving offspring (Bygott et al. 1979). Nevertheless, group living may be a burden when pride mates refrain from cooperative hunting and thus reducing net energy gain (Scheel and Packer 1991). Lions in prides often live in fission‐fusion organizations in which several subgroups are formed in a way that optimizes survival benefits including reproduction and foraging success (Packer et al. 1990). As described by the marginal value theorem, foragers will leave the group when the energy gains fall below their requirements or more profitable foraging is available in other groups and given perfect information about those groups (Davis et al. 2022). It is likely that per capita food intake is a proximate driver of fission–fusion dynamics in African lions. Group size has been shown to affect male lion feeding behavior and profitability (Scheel and Packer 1991; Funston et al. 1998). However, it is unclear how demographic structure affects the overall feeding dynamics within prides. In the Tarangire–Manyara ecosystem in Tanzania, lions feed in mixed demographic groups ranging from one to about 13 individuals (Gidna et al. 2014). This provides an opportunity to understand how sociological structures such as numbers of age/sex classes affect feeding dynamics and how foraging governs group composition and social organization of lion prides. In this study, we assess the effect of African lion feeding group demographic structure on per capita food intake of individual lions.

African lion prides are composed of varied demographic structures exhibiting hierarchy and age and sex distinct roles in cubs, subadults, adult females, and adult males (Mosser and Packer 2009; Packer and Pusey 1997). This social structure in prides is likely to influence competition patterns and feeding dynamics, making food unevenly distributed among pride members of different age/sex groups. We therefore, predict that prey size and group composition will affect the interaction patterns and per capita food intake of individual lions at carcasses. We hypothesize the following: (1) Prey body mass affects African lion feeding group composition at carcasses. (2) Prey size affects the interaction patterns of lions of different age/sex groups. (3) Feeding group composition affects per capita food intake.

## 2 Materials and Methods

### 2.1 Study Area Description

**FIGURE 2:**
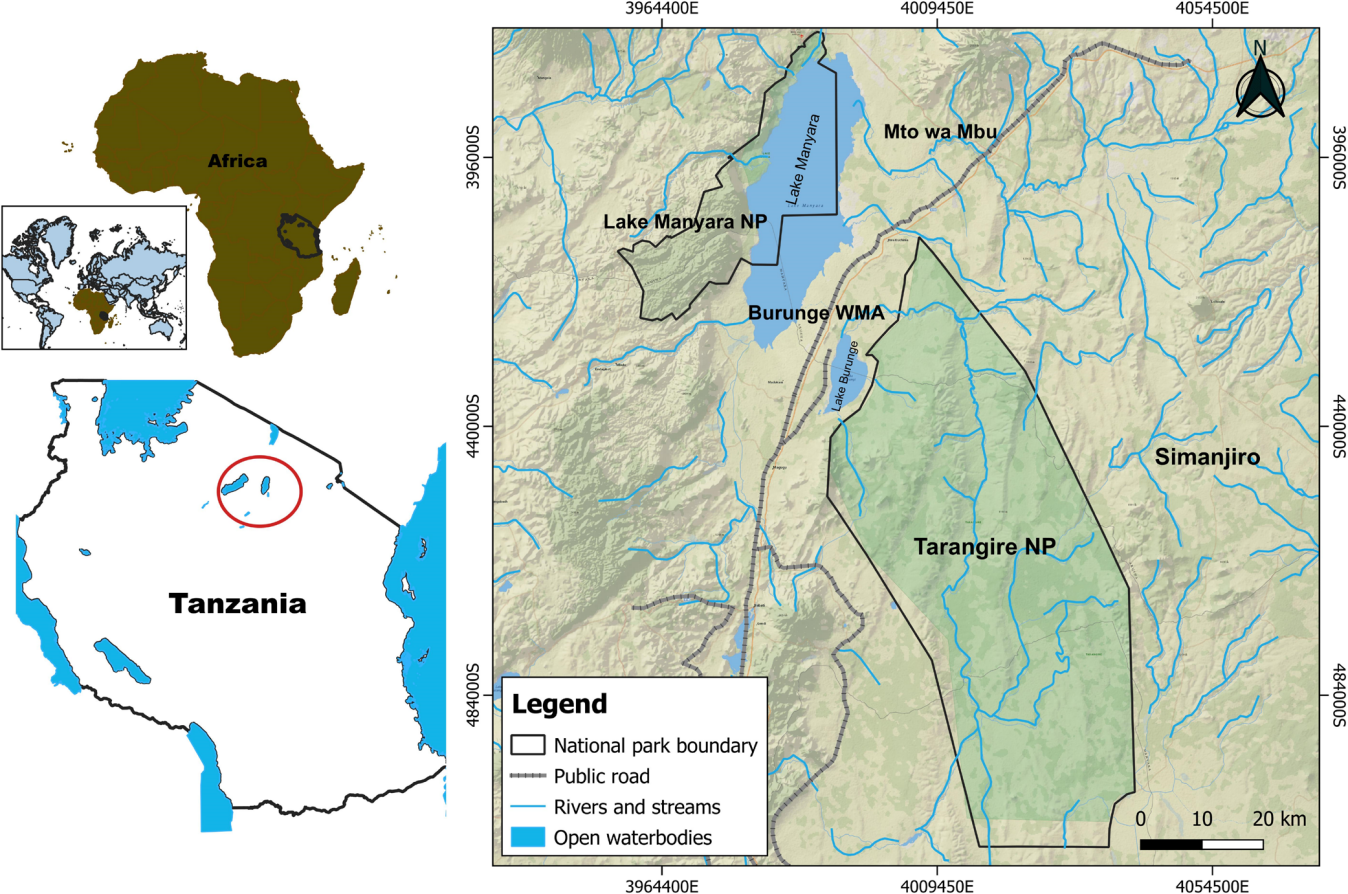
Map showing location of the study area.

This study was conducted in the Tarangire–Manyara ecosystem which covers 35,000 km^2^ (Kioko et al. 2013). The ecosystem is in northern Tanzania at an elevation ranging from 1000 to 2600 m above sea level. The ecosystem comprises a variety of core protected areas including Tarangire National Park, Lake Manyara National Park, and Mkungunero Game Reserve. Other protected areas which form the ecosystem include the Manyara Ranch, Lake Burunge, and Randilen WMAs (Wildlife Management Areas) and Game Controlled Areas (Mto wa mbu GCA, Lolkisale GCA) (Figure 2). WMAs are community lands set aside for the purpose of improving community‐based wildlife conservation while enhancing economic development and poverty reduction by granting ownership and management to local communities. GCAs are wildlife ranges outside of core protected areas designated for wildlife management. According to Tanzania Wildlife Conservation Act, some form of consumptive utilization such as licenced hunting, settlement and agriculture are allowed in both WMAs and GCAs (United Republic of Tanzania, 2009).

The study area vegetation forms a mosaic of ground water forests in parts of Lake Manyara National Park, woodland, bushland, shrubland, and open grasslands (Greenway and Vesey‐Fitzgerald 2016; Kioko et al. 2011). The main sources of water include Lake Manyara, Lake Burunge, and the Tarangire River. The surrounding land is dominated by rain‐fed agriculture and livestock grazing since a large portion of the communities are pastoralists (Galanti et al. 2006). The Tarangire–Manyara ecosystem is renowned for its diverse mammalian fauna, including African lions and other large predators like leopards ( *Panthera pardus* ), cheetahs ( *Acinonyx jubatus* ), spotted hyenas ( *Crocuta crocuta* ), striped hyena (*Hyena hyena*), and wild dog ( *Lycaon pictus* ) (Kiffner et al. 2022). Herbivore species include African bush elephant ( *Loxodonta africana* ), African buffalo ( *Syncerus caffer* ), Maasai giraffe (*Giraffa camelopardis*), wildebeest ( *Connochaetes taurinus* ), waterbuck ( *Kobus ellipsiprymnus* ), bushbuck ( *Tragelaphus scriptus* ), impala ( *Aepyceros melampus* ), Grant's gazelle ( *Nanger granti* ), zebra ( *Equus quagga* ), hartebeest ( *Alcelaphus buselaphus* ), eland (*Tragelaphus oryx*), and warthog ( *Phacochoerus africanus* ).

### 2.2 Data Collection

In this study, we used long‐term lion population monitoring data collected from 2004 to 2023 by the Tarangire Lion Project (TLP) with the support of the Maasai Steppe Carnivore Conservation Trust (MCCT). Radiotelemetry using VHF collars (Advanced Telemetry Systems Inc., model M2520B, Isanti, MN, USA) and GPS collars (Isanti, MN, USA) were used as the primary technique for long‐term monitoring and collecting information on lion demography, population estimates, movement patterns, and feeding and predator–prey interactions. Radio and/or GPS collars were attached to at least one individual lion from each pride, preferably females because they were usually permanent members of a pride (Packer and Pusey 1997). For each lion sighting, the following information was recorded: (i) the spatial location of the lions using GPS coordinates, (ii) individual identification based on unique whisker spots, (iii) group size, age, and sex of all lions present, and (iv) the time and date observations were recorded. A total of 4030 observations were recorded in 17 prides during this study. These observations were recorded by vehicle surveys conducted at least three times per month for every pride across the Tarangire–Manyara landscape. When lions were sighted with a kill, age‐sex class and species of all prey carcasses eaten by lions were recorded. Inspection of the kill site was also done to determine whether the prey was killed or scavenged (Gidna et al. 2014).

#### 2.2.1 Ethical Note

This study was conducted in compliance with the requirements of the United Republic of Tanzania under the TAWIRI‐COSTECH research clearance framework. Data were collected in the field involving a wild population of African lions managed in an in situ environment and so no specimen(s) was transported nor kept in captive facilities. GPS collaring adhered to the ethical guidelines provided by Tanzania Wildlife Research Institute (TAWIRI) and the legal framework provided by Tanzania Commission for Science and Technology (COSTECH) with research permit No: 2021‐298‐NA‐2019‐191.

#### 2.2.2 Feeding Group Demographic Structure

To determine the demographic structure of feeding groups, we used a combination of field observation data and demographic information for every individual lion ever sighted in the study area. For every feeding group, demographic information was recorded including the estimated birth dates and sexes of each individual. Individual lion age was calculated by subtracting the birth date from the observation date. We then categorized observed lions into cubs with age less than 2 years, subadults aged 2–4 years, and adults with more than 4 years of age (Schaller 1972; Smuts et al. 1980).

### 2.3 Data Analysis

#### 2.3.1 Effect of Prey Size on Feeding Group Demographic Structure and Interaction Patterns

We assessed the effect of prey size on African lion feeding group composition using multivariate generalized linear modeling, conducted in the “mvabund” package in R (Wang et al. 2012). We defined our response as counts of each age/sex at each carcass. We assumed numbers of individuals per class followed a Poisson distribution and used prey mass as the predictor. Furthermore, we tested the effect of prey size on interaction patterns of lions at carcasses by using probabilistic cooccurrence models between each pair of age/sex categories. We employed probabilistic cooccurrence analysis in presence–absence matrices indicating presence of at least one member of each age/sex group at a feeding event using the “cooccur” package Version 1.3 in R (Griffith et al. 2022). We further quantified the magnitudes of pairwise associations using effect sizes (Veech 2013) which was calculated as:

$$\text{Effect Size}=\frac{\text{Observed Cooccurrence}-\text{Expected Cooccurrence}\;}{\sqrt{\text{Expected Cooccurrence}}}$$

Effect sizes are standardized measures which quantify the strength and direction of variation from random expectation for each pair of age/sex groups. Positive values indicate preferential group pairs while negative values indicate pairs where avoidance would be expected. We used cooccurrence probabilities and effect sizes to evaluate the association of all age/sex category pairs across different prey sizes, including small (prey with more than 50 kg less than African lion adult body mass), medium (prey within 50 kg range of African lion adult body mass), and large (prey with more than 50 kg larger than African lion adult body mass).

#### 2.3.2 Estimation of Carcass Biomass Per Capita Intake

We used 312 confirmed lion‐hunted wild prey kills because we could not estimate the amount of biomass lions obtained from scavenged carcasses. This included observations made in 14 prides. For each carcass, we obtained prey species' body masses from adult body masses presented by Pacifici et al. (2013). We derived age‐based body masses for each prey carcass by considering whether the prey was a calf, yearling or adult (Radloff and Du Toit 2004). Calves and yearling body masses were derived as fractions of the adult body masses based on growth rates estimated by Schwartz and Hundertmark (1993) and Verme (1989). Predators do not consume the entire prey body mass (Sih 1980). For prey such as ungulates, anatomically edible body mass for carnivores is estimated to be about 65%–80% (Power 2003; Rapson and Bernard 2007). We assumed that lions do not consume the entire edible biomass due to the selective consumption of tissues (Domínguez‐Rodrigo 1999), water content of the edible biomass (Mattson 1997), and the presence of sympatric predators and scavengers that interact with lions at carcasses (Amorós et al. 2020; Hayward and Kerley 2008) which disrupt consumption by predators (Krofel et al. 2012). We therefore, estimated the available biomass of prey ($B_{\text{Prey}}$) for all carcasses at 50% of the prey mass as estimated by Blumenschine and Caro (1986). When half of the meat had been consumed at the time of observation, we divided the biomass by two, while we divided by four when only one‐quarter remained. This proportion is subjective because the amount of carcass left may vary based on the feeding selectivity of lions. For that reason, these fractions form a necessary assumption for comparing the amounts of prey observed.

From the available biomass in each carcass, we estimated per capita biomass intake for each lion in the feeding group based on their age/sex group's consumption rates. Consumption was considered to be one third and two thirds as much as adult females for cubs and subadults respectively, while male lion consumption was assumed to be 1.5 times that of adult females (Funston et al. 1998; Packer et al. 1990). We used these ratios to estimate per capita food intake for cubs, subadults, adult females, and adult males at each carcass. Firstly, we estimated the relative consumption (RC) by cubs (i), subadults (ii), adult females (iii), and adult males (iv) of each carcass as a proportion of the biomass eaten by every age‐sex group based on their numbers ($N_{\text{cubs}},N_{\text{subadults}},\;{N_{\text{Adult}}}_{♀},$ and ${N_{\text{Adult}}}_{♂}$ respectively). Then the total relative consumption ($\text{Tota}l_{\mathrm{RC}}$) was calculated as the sum of all Relative Consumptions for each age‐sex group for each carcass (v), followed by estimation of the total biomass ($TB_{\text{Cubs}}$, $TB_{\text{Subadult}}$, ${{\mathrm{TB}}_{\text{Adult}}}_{♀}$, and ${{\mathrm{TB}}_{\text{Adult}}}_{♂}$) consumed by each group (vi‐ix). Finally, we divided the total biomass consumed by each age‐sex group by the number of individuals in respective groups (x‐xiii) to estimate per capita consumption ($PC_{\text{Cubs}}$, $PC_{\text{Subadults}}$, ${{\mathrm{PC}}_{\text{Adult}}}_{♀}$, and ${{\mathrm{PC}}_{\text{Adult}}}_{♂}$).

(1) $$RC_{\text{Cubs}}=\frac{1}{3}\times N_{\text{cubs}}$$

(2) $$RC_{\text{Subadults}}=\frac{2}{3}\times N_{\text{subadults}}$$

(3) $$R{C_{\text{Adult}}}_{♀}=1\times {N_{\text{Adult}}}_{♀}$$

(4) $$R{C_{\text{Adult}}}_{♂}=1.5\times {N_{\text{Adult}}}_{♂}$$

(5) $$\text{Tota}l_{\mathrm{RC}}=RC_{\text{Cubs}}+RC_{\text{Subadults}}+R{C_{\text{Adult}}}_{♀}+R{C_{\text{Adult}}}_{♂}$$

(6) $$TB_{\text{Cubs}}=\frac{RC_{\text{Cubs}}}{\text{Tota}l_{\mathrm{RC}}}\times B_{\text{Prey}}$$

(7) $$TB_{\text{Subadult}}=\frac{RC_{\text{Subadults}}}{\text{Tota}l_{\mathrm{RC}}}\times B_{\text{Prey}}$$

(8) $${{\mathrm{TB}}_{\text{Adult}}}_{♀}=\frac{R{C_{\text{Adult}}}_{♀}}{\text{Tota}l_{\mathrm{RC}}}\times B_{\text{Prey}}$$

(9) $${{\mathrm{TB}}_{\text{Adult}}}_{♂}=\frac{{{\mathrm{RC}}_{\text{Adult}}}_{♂}}{\text{Tota}l_{\mathrm{RC}}}\times B_{\text{Prey}}$$

(10) $$PC_{\text{Cubs}}=\frac{TB_{\text{Cubs}}}{N_{\text{cubs}}}=\frac{\frac{1}{3}\times N_{\text{cubs}}\times B_{\text{Prey}}}{\text{Tota}l_{\mathrm{RC}}}\times \frac{1}{N_{\text{cubs}}}=\frac{\frac{1}{3}\;\times B_{\text{Prey}}}{\text{Tota}l_{\mathrm{RC}}}$$

(11) $$PC_{\text{Subadults}}=\frac{TB_{\text{Subadults}}}{N_{\text{Subadults}}}=\frac{\frac{2}{3}\times N_{\text{Subadults}}\times B_{\text{Prey}}}{\text{Tota}l_{\mathrm{RC}}}\times \frac{1}{N_{\text{Subadults}}}=\frac{\frac{2}{3}\;\times B_{\text{Prey}}}{\text{Tota}l_{\mathrm{RC}}}$$

(12) $${{\mathrm{PC}}_{\text{Adult}}}_{♀}=\;\frac{{{\mathrm{TB}}_{\text{Adult}}}_{♀}}{{N_{\text{Adult}}}_{♀}}=\frac{1\times {N_{\text{Adult}}}_{♀}\times B_{\text{Prey}}}{\text{Tota}l_{\mathrm{RC}}}\times \frac{1}{{N_{\text{Adult}}}_{♀}}=\frac{1\times B_{\text{Prey}}}{\text{Tota}l_{\mathrm{RC}}}$$

(13) $${{\mathrm{PC}}_{\text{Adult}}}_{♂}=\frac{{{\mathrm{TB}}_{\text{Adult}}}_{♂}}{{N_{\text{Adult}}}_{♂}}=\frac{1.5\times {N_{\text{Adult}}}_{♂}\times B_{\text{Prey}}}{\text{Tota}l_{\mathrm{RC}}}\times \frac{1}{{N_{\text{Adult}}}_{♂}}=\frac{1.5\times B_{\text{Prey}}}{\text{Tota}l_{\mathrm{RC}}}$$

For each age‐sex group's per capita intake at a carcass, we fitted Generalized Additive Models (GAMs) of quasipoisson family with a log link function using the “mgcv” package Version 1.9–1 in R (Wood 2023). Per capita biomass from a carcass for each age‐sex class individual was used as the response variable and number of cubs, subadults, adult males, and adult females as predictor variables for each carcass (Table S1). For each age‐sex category, we incrementally constructed sets of models. Firstly, we developed single‐predictor models with each of the four predictor variables, then we created more complex additive models using all possible combinations of the predictors, and lastly models with two‐way interactions between the four predictors were constructed to evaluate individual demographic group effects, joint effects, as well as pairwise interaction effects of cooperative foraging (Table S2). To compare model fit, we assessed the performance of each model based on Generalized Cross‐Validation (GCV) in which the lowest value indicates the best fit (Wood 2001). We then used the best model to explain the relationship between the variables.

## 3 Results

**FIGURE 3:**
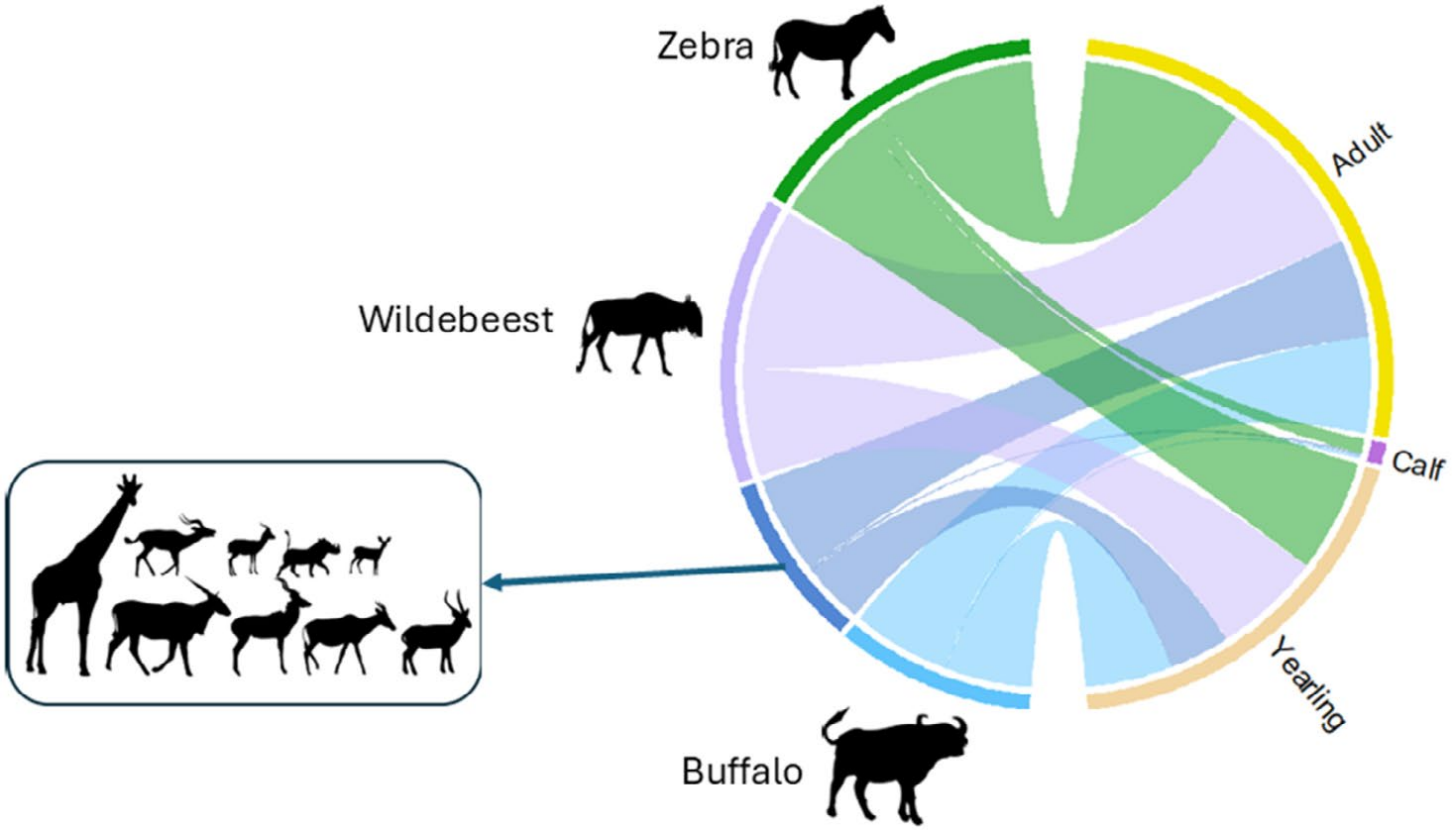
Observed African lion wild ungulate prey kills age‐class distribution. Each ribbon connects observed species killed (left) to the age classes (right). The width of each ribbon represents the proportional contribution to the number of observed kills (*n* = 312). Vector graphics from phylopic.org.

**FIGURE 4:**
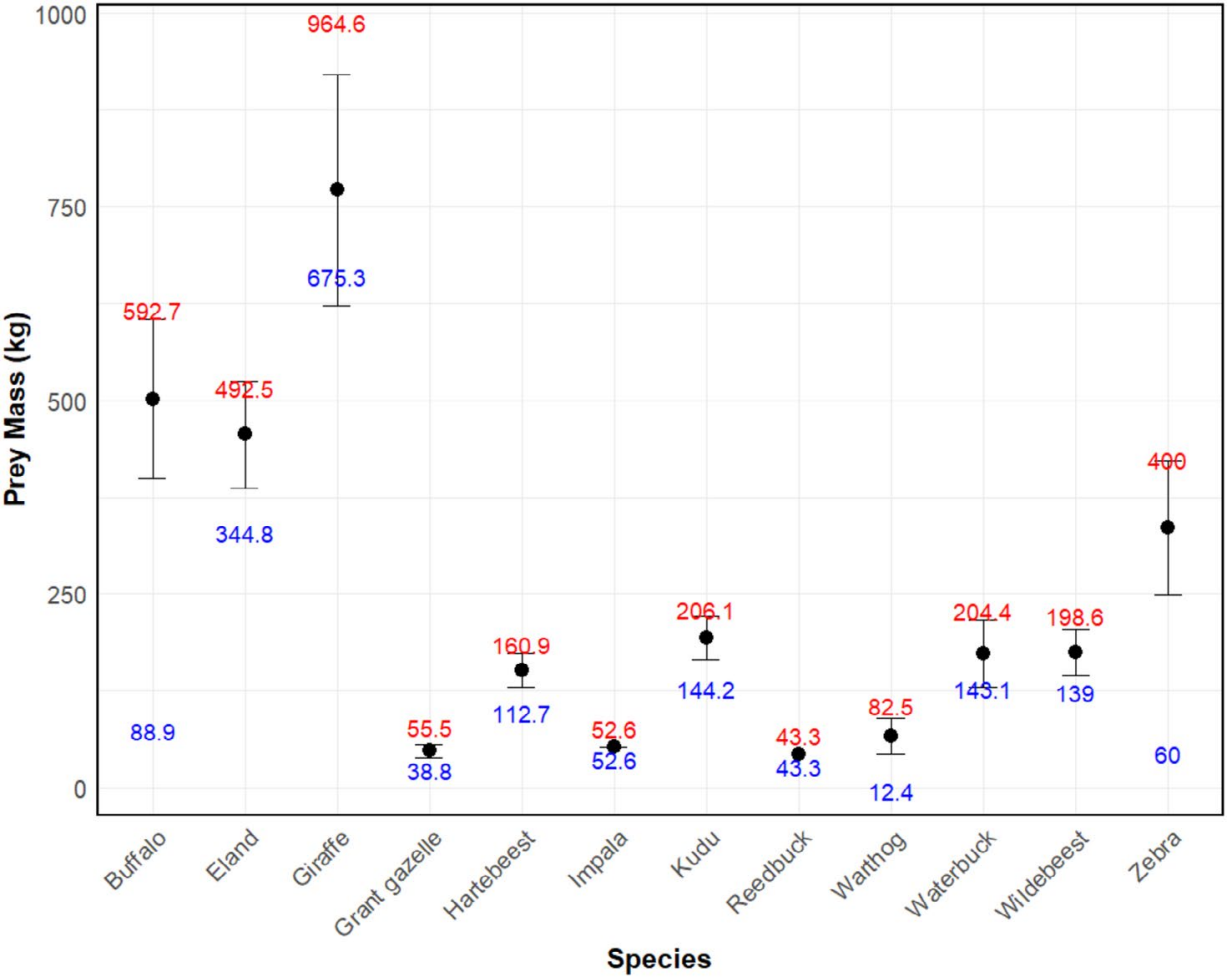
Estimated mean prey masses of the observed prey kills. Points represent the average mass for each prey species, error bars represent standard deviation (±SD). Labels represent maximum (red) and minimum (blue) estimated body masses for all prey species.

Our results showed that 12 wild ungulates contributed to the diet of the African lion population in the Tarangire–Manyara ecosystem. Zebra (31.7%, *n* = 99), wildebeest (29.8%, *n* = 93), and buffalo (20.5%, *n* = 64) dominated the African lion diet. Other prey included nine species which were recorded infrequently (18%, *n* = 56). Most ungulates preyed upon were adults (57.1%, *n* = 178) and yearlings (40.7%, *n* = 127, Figure 3). The kills represented a wide range of prey sizes, ranging from 12.4 kg to 964.6 kg (mean = 299.8 kg, median = 280 kg). Giraffe (mean = 772 kg) and buffalo (mean = 502 kg) were the largest prey species, while Grant's gazelle and reedbuck were the smallest, with mean weight of less than 50 kg (Figure 4). Most kills were for large prey (74.4%, *n* = 232), while the remaining represented both small and medium prey (14.1%, *n* = 44, and 11.5%, *n* = 36, respectively).

**FIGURE 5:**
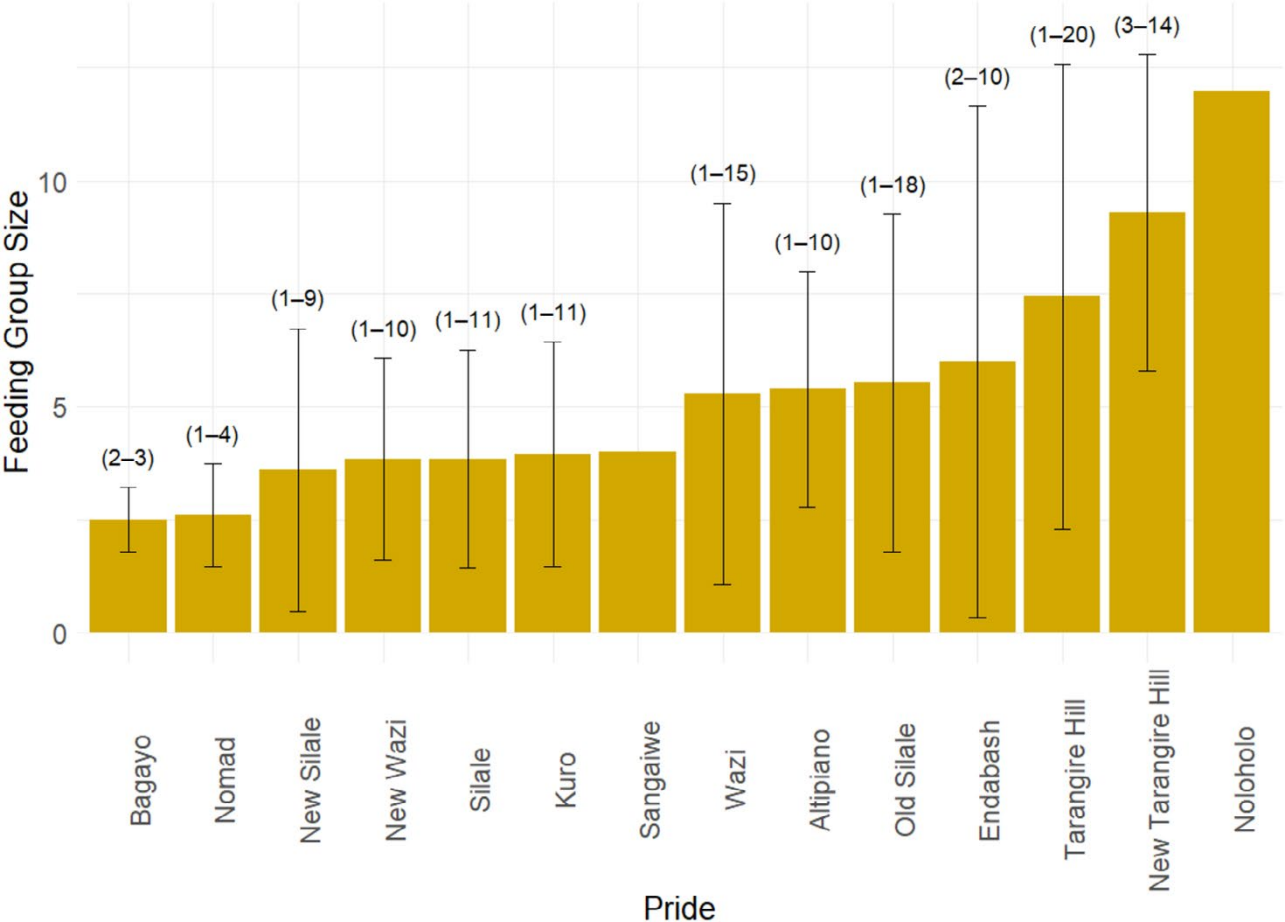
Mean feeding group sizes in different African lion prides in the Tarangire–Manyara ecosystem. Error bars represent variability (±SD). Labels on bars represent the range of observed feeding group sizes for each pride (Noloholo and Sangaiwe, *n* = 1).

**FIGURE 6:**
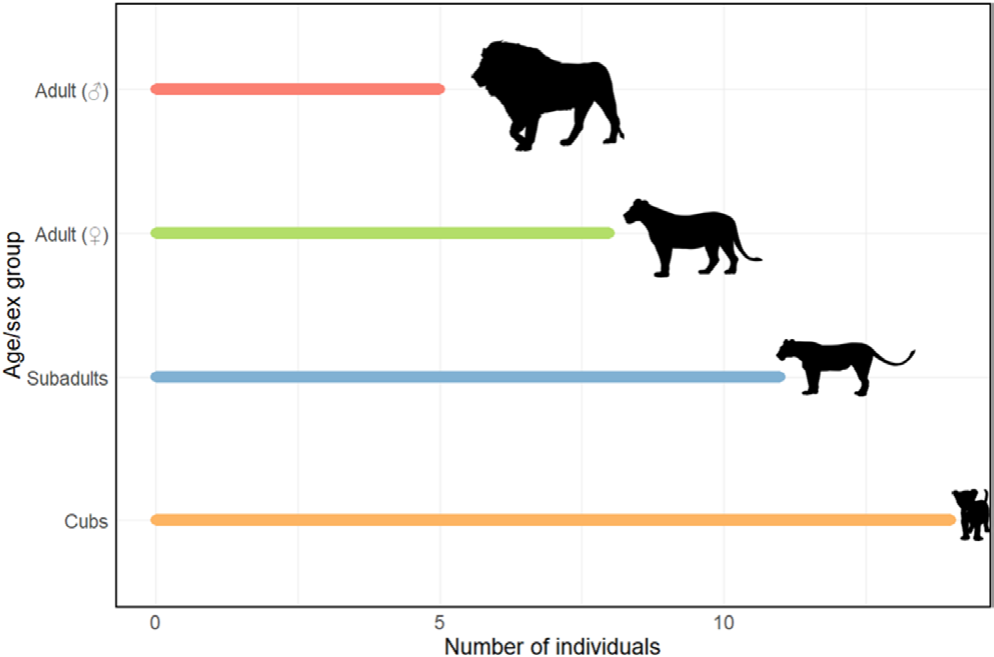
Range of number of individuals for each age/sex class in African lion prides for observed feeding groups. Vector graphics from phylopic.org.

**FIGURE 7:**
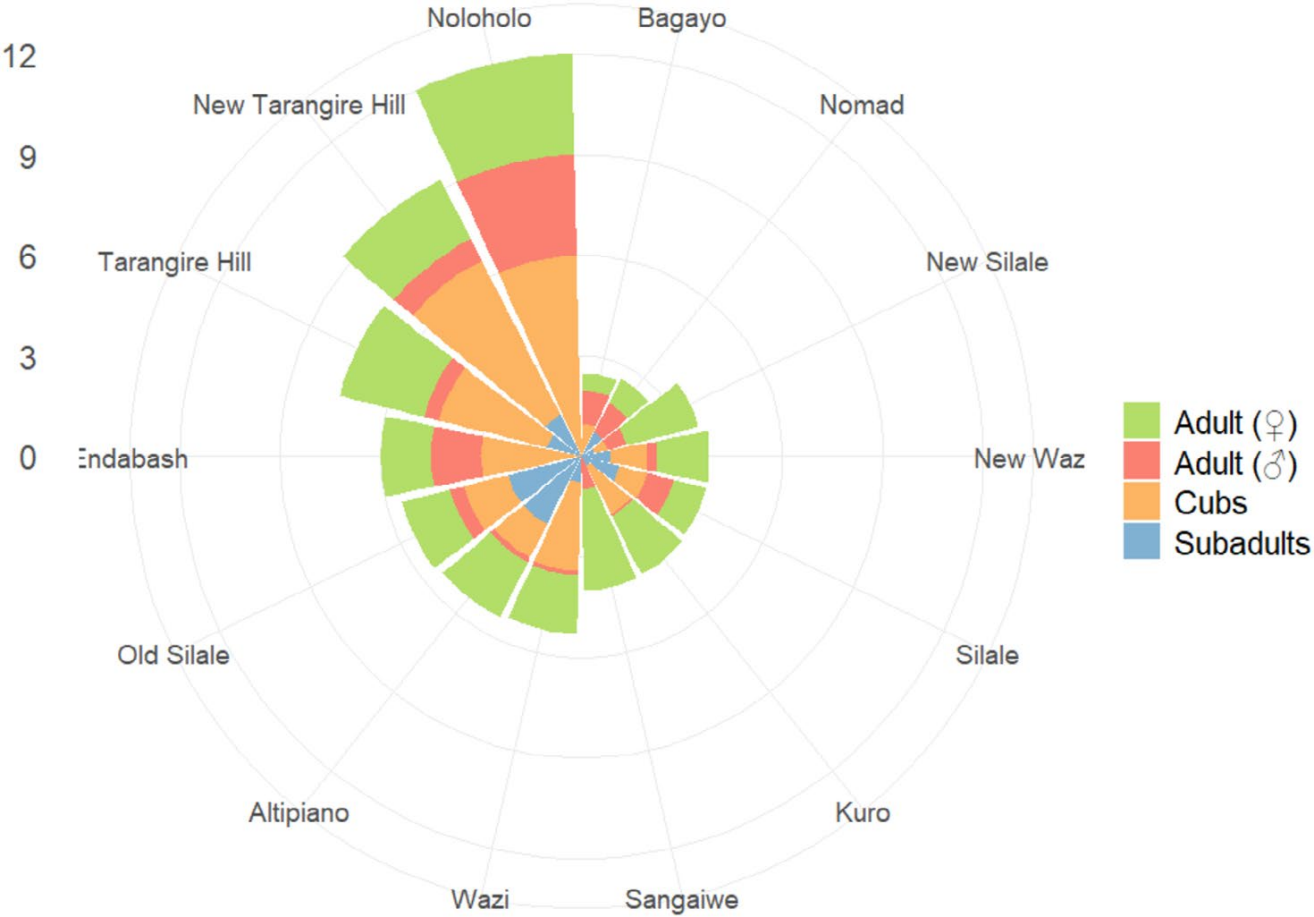
Average number of individuals observed in feeding events for all demographic groups across African lion prides in the Tarangire–Manyara ecosystem. Each segment indicates the average number of observed individuals in feeding events for each pride, whereas bar heights represent the average numbers of cubs, subadults, adult females, and adult males.

Most feeding events were observed in the Tarangire hill and Altipiano prides (97 and 62 observations, respectively), with the least in the Bagayo, Endabash, Noloholo, and Sangaiwe prides (1–2 observations). Feeding group sizes varied in observations and across prides. New Tarangire and Tarangire prides had notably high average group sizes, and the Tarangire pride showed the widest group size range, with up to 20 individuals in a feeding event. Although the Noloholo pride had the highest average feeding group size, this was based on only one observation with 12 feeding individuals (Figure 5). Overall, the number of each age/sex group varied across feeding events and was highest for cubs (up to 14) and low for adult males (maximum of 5 individuals) (Figure 6). Average numbers of each age/sex category also varied across prides (Figure 7).

**FIGURE 8:**
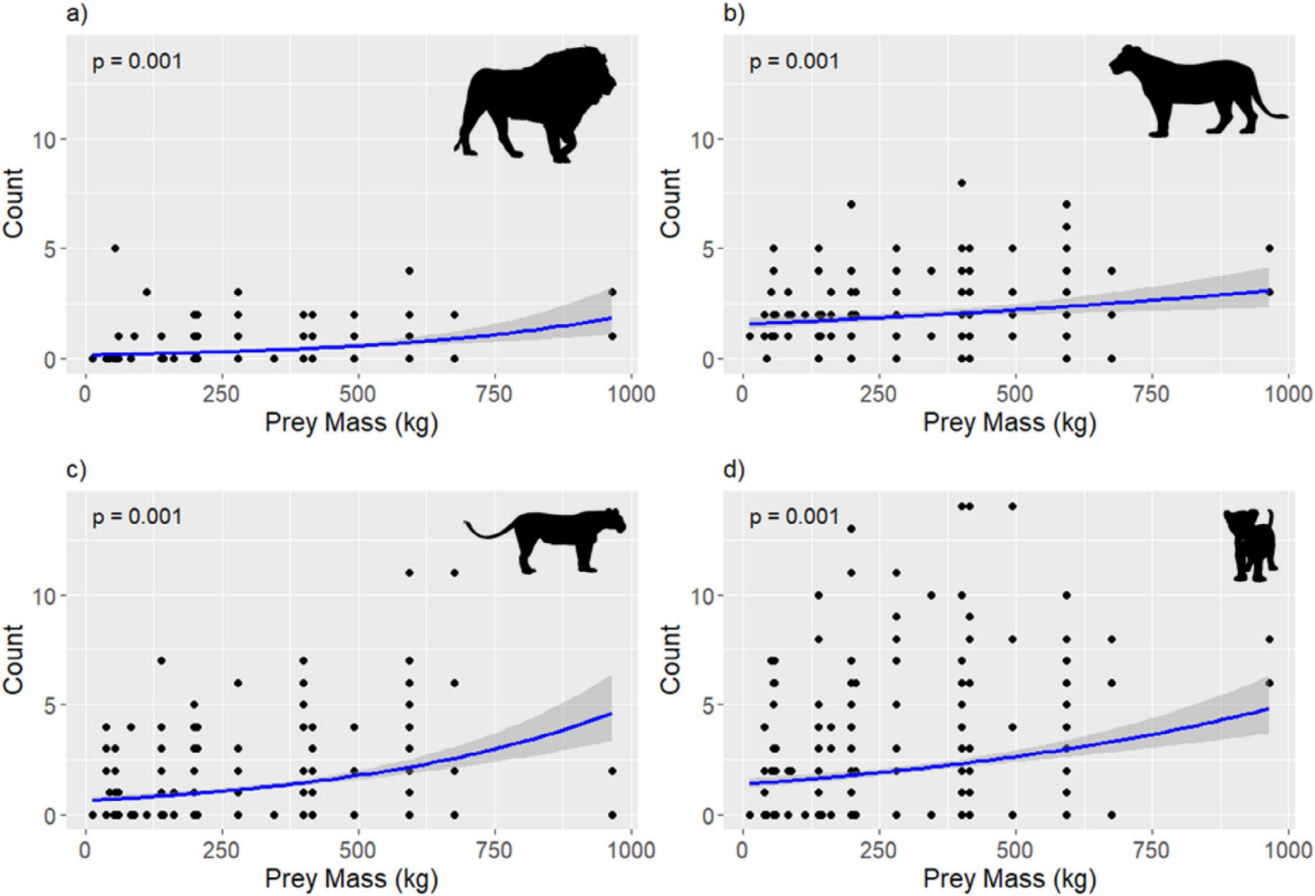
Relationship between prey mass (kg) and each demographic group composition in terms of number of (a) adult males, (b) adult females, (c) subadults, and (d) cubs. Figures correspond to multivariate generalized linear model indicating an effect of prey size on African lion feeding group demographic structure. Vector graphics from phylopic.org.

**FIGURE 9:**
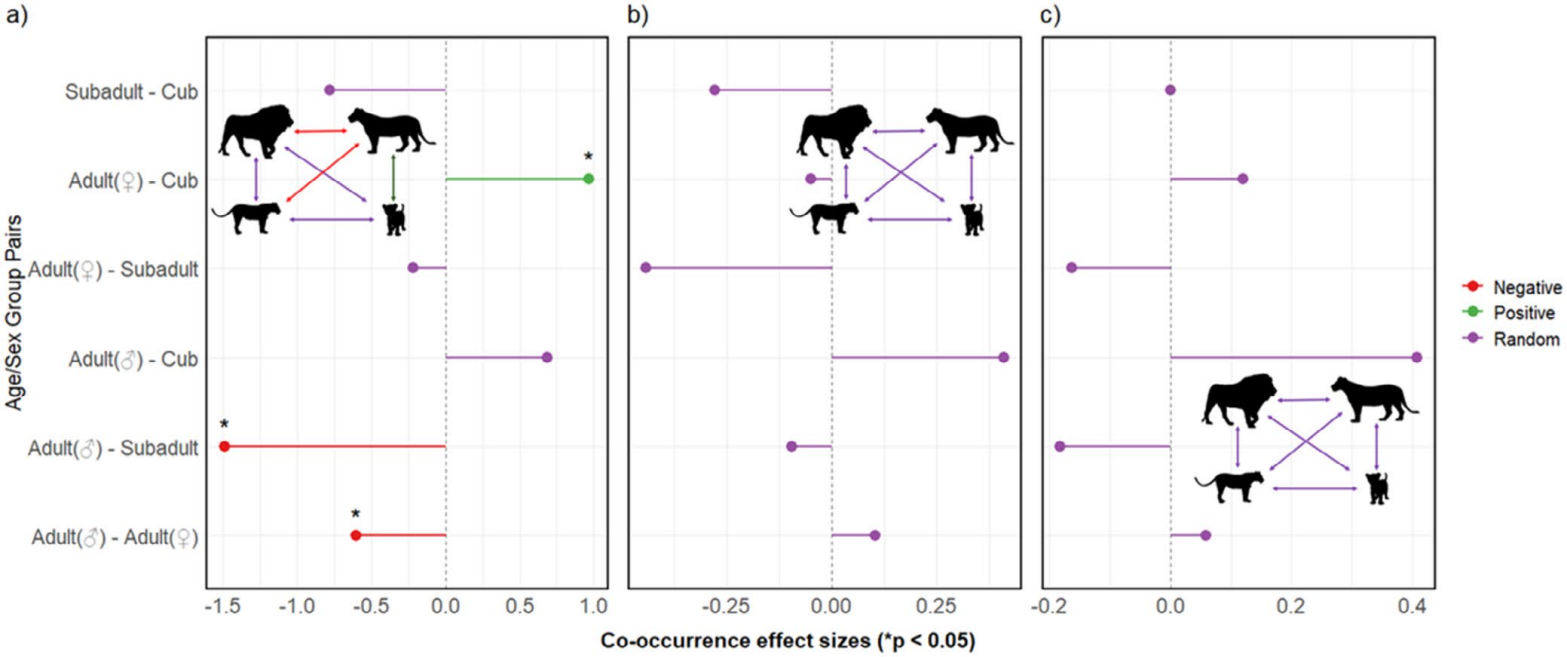
Standardized effect sizes for pairwise co‐occurrences between African lion age/sex class pairs across (a) large, (b) medium, and (c) small‐sized prey. Each point represents direction and strength of association between each demographic group pair. Arrows between age/sex class individuals represent association between species pairs (negative [red], positive [green], or random [purple]). Asterisks (*) denote statistically significant (non‐random) associations (*p* < 0.05). Vector graphics from phylopic.org.

Our results showed that prey mass has a significant effect on feeding group composition (Wald test statistic = 11.79, *p* = 0.001). Prey mass was positively associated with the numbers of all demographic groups, which show increased demographically diverse groups (Figure 8). Varied interaction patterns at carcasses were shown, with distinct associations across different prey sizes. Most age/sex category pair interactions were random (83.33%, *n* = 15), while three pairs' cooccurrence patterns deviated from random. For large prey, adult females had a strong positive association with cubs (effect Size = 0.969, *p* < 0.0001), while adult males had low co‐occurrences with adult females and subadults (effect sizes = −0.602 and −1.492, respectively; *p* < 0.05). All age/sex category pairs were random at small and medium‐sized prey (*p* > 0.05, Table 1 and Figure 9).

**TABLE 1:** Cooccurrence probability table for age/sex group pairs for different prey sizes. *p*_lt_ and *p*_gt_ are the probabilities of observing fewer and greater co‐occurrences, respectively, than expected under a null model.

Age/sex category pair	Cooccurrence probability	Effect size	*p*_lt_	*p*_gt_
**Large‐sized prey**				
Adult males–Adult females	0.298	−0.602	0.021	0.993
Adult males–Subadults	0.150	−1.492	0.009	0.996
Adult males–Cubs	0.163	0.683	0.903	0.153
Adult females–Subadults	0.406	−0.216	0.239	0.873
Adult females–Cubs	0.441	0.969	1.000	0.00001
Subadults–Cubs	0.222	−0.779	0.089	0.946
**Medium‐sized prey**				
Adult males–Adult females	0.087	0.103	1.000	0.825
Adult males–Subadults	0.025	−0.095	0.70335	0.735
Adult males–Cubs	0.035	0.408	0.84722	0.504
Adult females–Subadults	0.260	−0.442	0.06977	1.000
Adult females–Cubs	0.369	−0.049	0.62896	0.856
Subadults–Cubs	0.105	−0.279	0.46737	0.783
**Small‐sized prey**				
Adult males–Adult females	0.081	0.059	1.000	0.917
Adult males–Subadults	0.032	−0.183	0.66863	0.784
Adult males–Cubs	0.042	0.408	0.88571	0.500
Adult females–Subadults	0.378	−0.163	0.38889	1.000
Adult females–Cubs	0.486	0.119	1.000	0.500
Subadults–Cubs	0.194	0.000	0.63339	0.633

**FIGURE 10:**
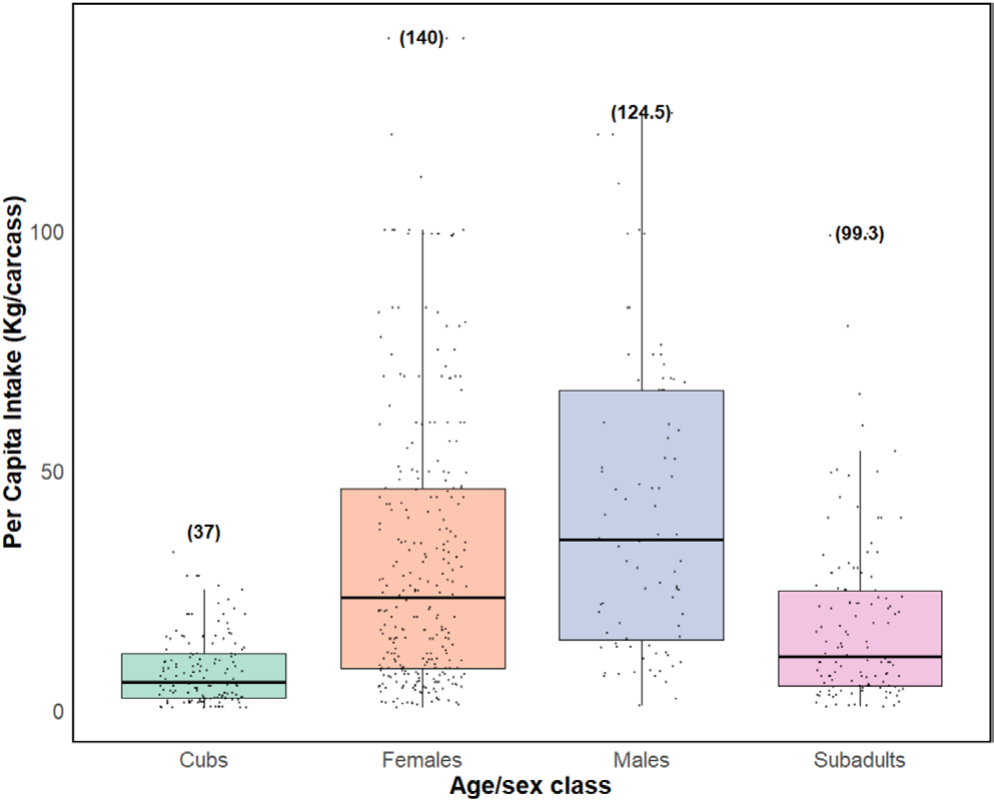
Per capita food intake at carcasses among African lion age/sex classes. Horizontal lines represent median values for each age/sex class; data points represent distribution of per capita food intakes. Values written above boxes indicate maximum per capita intakes for each age/sex class.

Per capita food intake at carcasses varied among the age/sex class groups. Adult males had the highest mean per capita food intake (mean = 42.7 kg, SD = 32.2, range = 0.83–124.0 kg), although the highest per capita intake values were observed for adult females (mean = 32.1 kg, SD = 29.6, range = 0.55–140.0 kg), followed by subadults (mean = 18.5 kg, SD = 19.5, range = 0.69–99.3 kg) and cubs (mean = 8.36 kg, SD = 7.34, range = 0.41–37.0 kg). Cubs had the lowest per capita food intake (Figure 10). Results indicate varied associations between each demographic group's per capita intake and numbers of lions of different age/sex classes within feeding groups. Cub per capita food intake additively decreased with an increase in the number of cubs and subadults. For subadults and adult females, per capita food intakes were best explained by the two‐way interaction of predictors, while adult male per capita food intake was best explained by an additive effect of adult males and adult female lions (Table S3).

**FIGURE 11:**
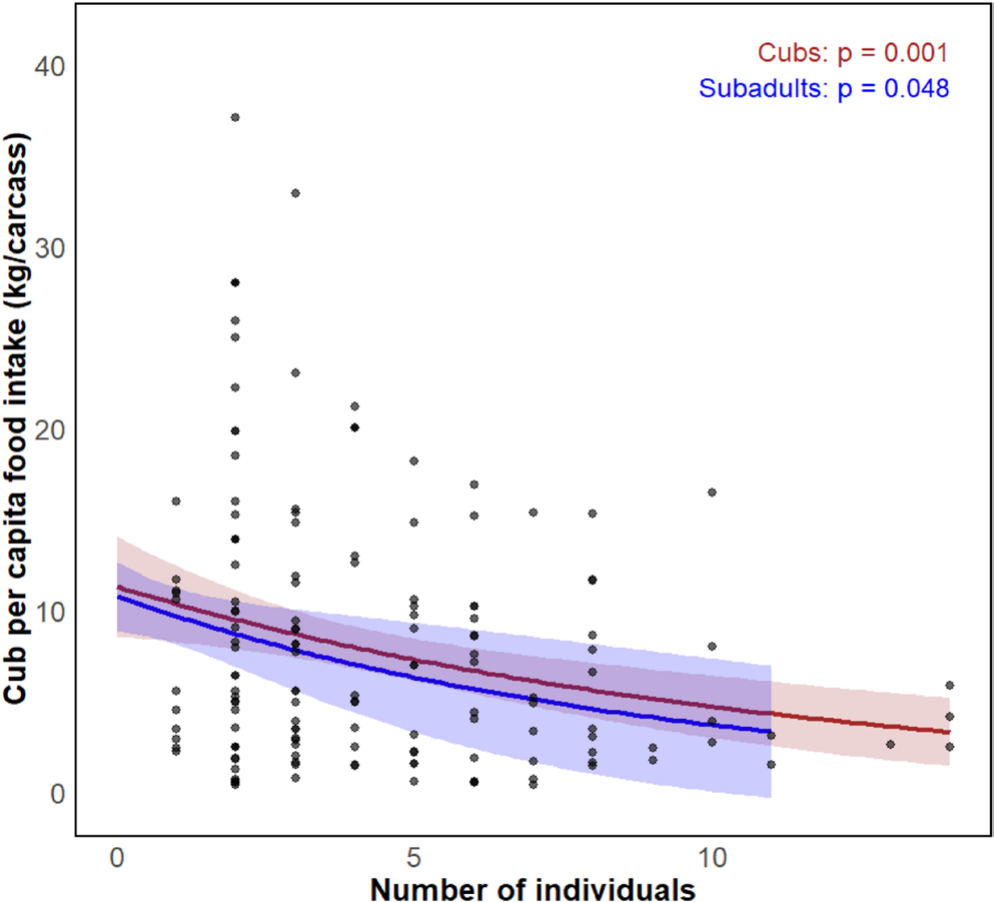
Relationship between feeding group demographic structure (number of cubs [brown] and subadults [blue]) and cub per capita food intake predicted by additive model of cubs and subadults.

**FIGURE 12:**
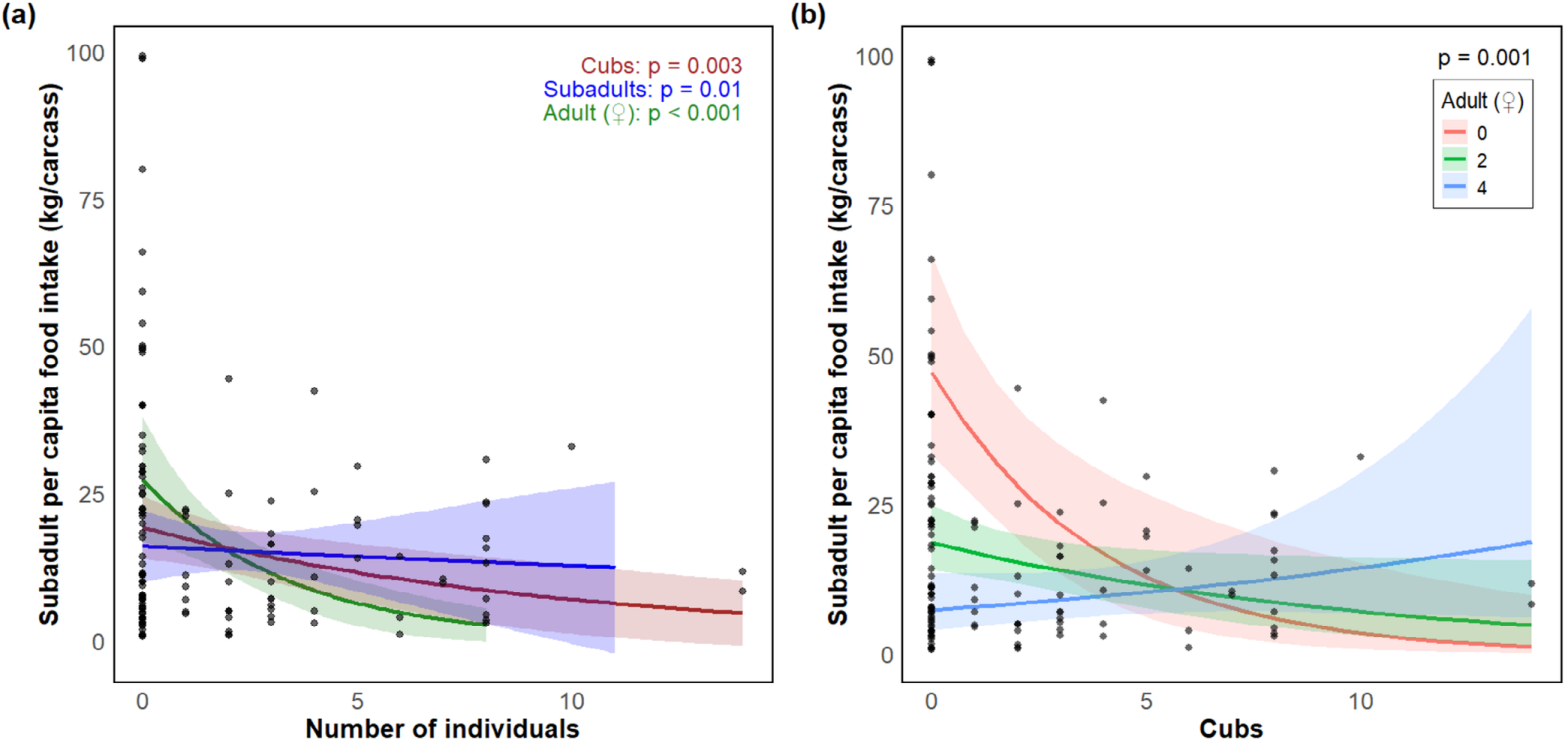
Relationship between feeding group demographic structure and subadult per capita food intakes. (a) Main effects of number of cubs (brown), subadults (blue), and adult females (green); (b) interaction effects of number of cubs and number of adult females predicted by two‐way interaction model.

**FIGURE 13:**
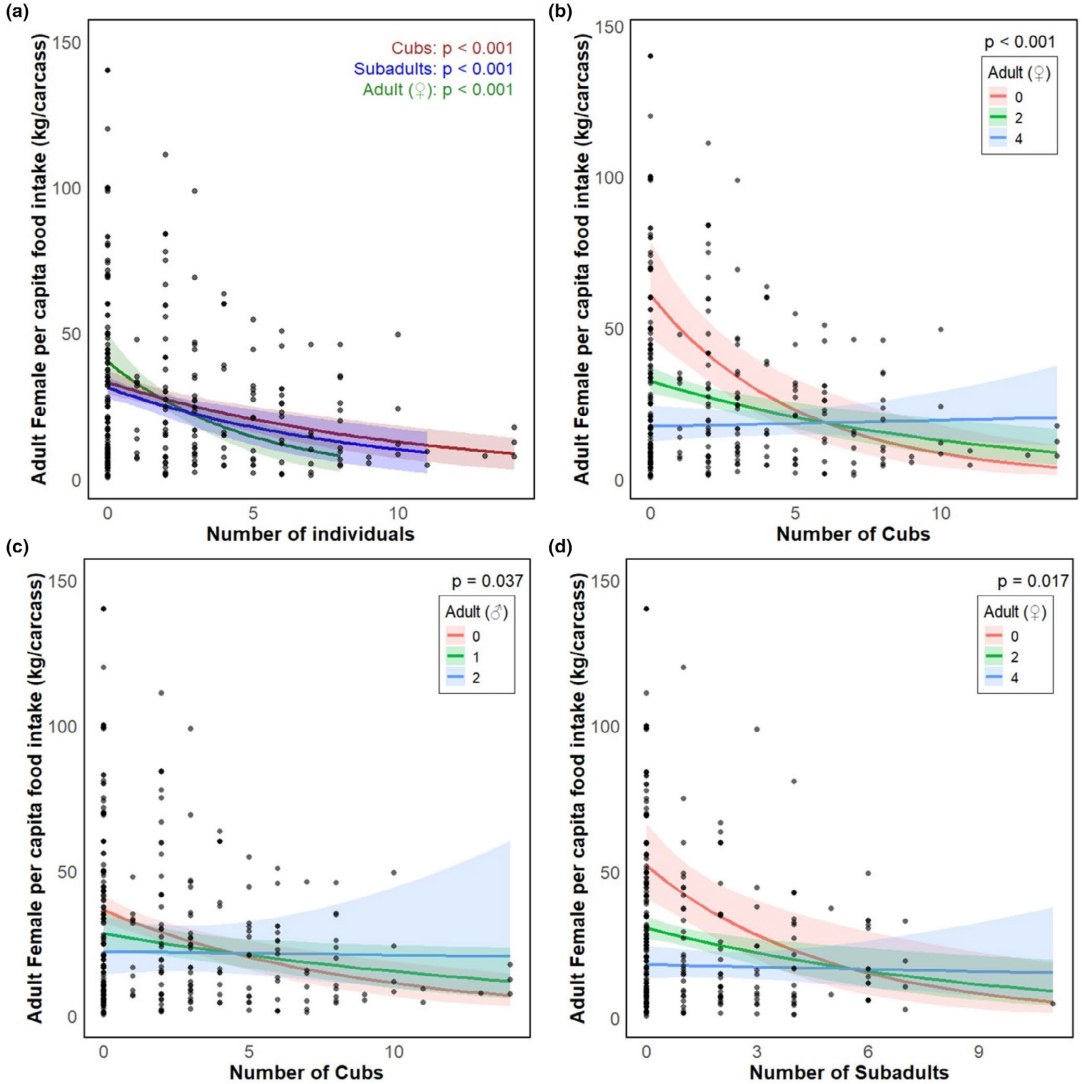
Relationship between feeding group demographic structure and adult female per capita food intakes. (a) Main effects of number of cubs (brown), subadults (blue), and adult females (green); (b) interaction effects of number of cubs and adult females; (c) Interaction effects of number of cubs and adult males, and (d) interaction effects of number of subadults and adult females predicted by two‐way interaction model.

**FIGURE 14:**
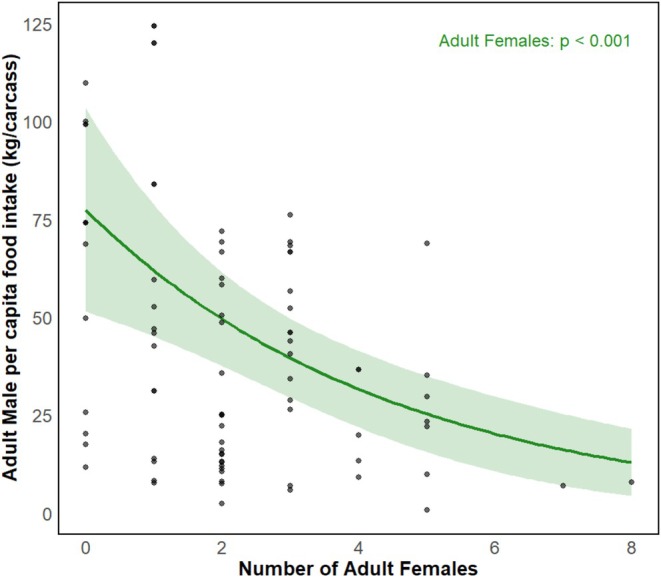
Relationship between feeding group demographic structure (number of adult females) and adult male per capita food intake predicted by additive model.

Cub per capita intake declined with increased numbers of cubs (*p* = 0.001) and subadults (*p =* 0.048) (Table 2 and Figure 11). Subadult per capita intake was reduced by the increase in the number of cubs (*p* < 0.01) although it increased when feeding together with cubs and adult females (*p* < 0.01), and also decreased with the increase in the number of subadults (*p* < 0.01) (Table 2 and Figure 12). Subadult per capita intake was similarly negatively associated with the increase in the number of females (*p* < 0.001) (Table 2 and Figure 12). For adult females, per capita food intake decreased with increases in the number of cubs (*p* < 0.001), although it increased in the presence of additional adult females (*p* < 0.001) or males (*p* < 0.05) (Table 2 and Figure 13). Adult female per capita intake was also reduced with increased numbers of subadults (*p* < 0.001) but also increased with the number of adult females (*p* < 0.05). Adult female per capita intake also decreased with increases in the number of adult females (*p* < 0.001) (Table 2 and Figure 13). Adult male per capita intake decreased with the increase in the number of adult females (*p* < 0.001, Table 2 and Figure 14).

**TABLE 2:** Summary of the most supported models, showing effect of predictors on per capita intake for each age/sex group.

Focal group	Predictors	Estimate	Std. error	*t* value	*p*
Cubs	Cubs	−0.087	0.027	−3.247	0.001
	Subadults	−0.107	0.053	−1.995	0.048
Subadults	Cubs	−0.305	0.099	−3.072	0.003
	Subadults	−0.167	0.063	−2.635	0.0095
	Adult females	−0.534	0.124	−4.32	< 0.001
	Adult males	−0.272	0.361	−0.753	0.453
	Cubs × Subadults	0.029	0.034	0.851	0.396
	Cubs × Adult females	0.082	0.025	3.32	0.001
	Cubs × Adult males	0.023	0.046	0.498	0.619
	Subadults × Adult females	0.04	0.027	1.492	0.138
	Subadults × Adult males	0.015	0.106	0.143	0.886
	Adult females × Adult males	0.054	0.122	0.444	0.658
Adult females	Cubs	−0.248	0.042	−5.881	< 0.001
	Subadults	−0.268	0.054	−4.976	< 0.001
	Adult females	−0.378	0.08	−4.739	< 0.001
	Adult males	−0.326	0.195	−1.675	0.095
	Cubs × Subadults	0.024	0.017	1.385	0.167
	Cubs × Adult females	0.052	0.013	3.875	0.001
	Cubs × Adult males	0.056	0.026	2.101	0.037
	Subadults × Adult females	0.047	0.02	2.402	0.017
Adult males	Subadults × Adult males	0.037	0.061	0.606	0.545
	Adult females × Adult males	0.017	0.08	0.214	0.831
	Adult females	−0.224	0.054	−4.112	< 0.001
	Adult males	−0.159	0.101	−1.572	0.12

## 4 Discussion

Our results showed that African lions in the Tarangire–Manyara ecosystem have a catholic diet, feeding on a variety of wild prey types. The dominant prey species recorded in our study are similar to those reported by Makacha and Schaller (1969) in the similar ecosystem and those in other parts of Africa. For example, in South Africa, lions were reported to feed on both the same prey types and other prey species that were not observed in our study, like nyala ( *Tragelaphus angasii* ) and white rhinoceros ( *Ceratotherium simum* ) (Barnardo et al. 2020). In Central and West Africa, additional species such as baboon and kob have been reported (Bauer et al. 2008). These results support the idea that lions are opportunistic predators.

Prey size showed a significant effect on African lion feeding group composition and was associated with the number of lion individuals of all demographic groups at a kill. The observed increase in the number of individuals with an increase in prey size is consistent with the fact that larger prey provide higher energetic returns. High energetic returns at large prey may facilitate group cohesion by reducing competition within large prides. Furthermore, our results also imply that African lion group organization is driven by resource availability, which may in turn shape group composition. This variation in number may be a behavioral flexibility that serves to minimize conflict while maximizing food intake. In large social carnivores, group size is known to be a key attribute to hunting success, not only in African lions but also other carnivores such as African wild dogs (Creel and Creel 1995).

There was also variation in the association patterns of lions between different prey sizes. Random association between all age/sex category pairs in small‐ and medium‐sized prey indicates uncoordinated cooccurrence among pride members. This suggests that when food is limited, pride member coexistence at carcasses is opportunistic, with no structured interaction patterns, perhaps due to the influence of dominant pride members. Conversely, nonrandom associations observed at large‐sized prey reflect feeding and social dynamics occurring when feeding within prides. Positive association between adult females and cubs suggests that rearing females actively hunt large prey to meet the energy requirements of them and their cubs, or to facilitate vertical social transmission of hunting skills, which is common throughout the animal kingdom (Wild et al. 2020). This may also be attributed to communal rearing of cubs, which allows females that are not rearing young to hunt and have some form of tolerance for younger individuals, especially when food is abundant (Packer and Pusey 1997). The negative association between adult males and adult females and subadults may be attributed to competition and segregation strategies between dominant individuals (adult males) which could displace subordinate members. Dominance hierarchy social systems are common in large carnivores and play critical roles in the organization of group living (Clapham et al. 2012; Sands and Creel 2004). Similarly, subadults and adult females may control adult male participation and food access through avoidance and reduced aggression associated with group membership and prey size.

Studies on foraging show high benefits obtained by social carnivores compared to solitary hunters, with higher net food gain for individuals in larger groups (Eklöv 1992; Vucetich et al. 2004). These benefits may be attributed to cooperative hunting, which outweighs the trade‐offs of hunting alone, especially in terms of prey capture success (Hector 1986; MacNulty et al. 2014) and decreased losses from competitors (Carbone et al. 1997). For predators, energy return is a net gain relative to the time and effort invested in prey capture (Benoit‐Bird 2004; Elimelech and Pinshow 2008; Higgins and Buskirk 1992; McInnes et al. 2017), handling (Hoyle and Keast 1987; Kaspari 1990; Nordlund and Morrison 1990; Okuyama 2010; Schreiber and Vejdani 2006), and consumption (Langerhans et al. 2021). Feeding in groups is associated with competition, aggression, and refraining from hunting, especially when the numbers of predators are too large (Packer and Ruttan 1988). Social predators like lions not only benefit from cooperative foraging through increased prey capture success, but also from defending resourceful territories, increased survival of offspring, as well as creating chances for learning, essential for species survival (Krebs and Inman 1992; Mosser and Packer 2009).

A reduced per capita intake for cubs with an increased number of cubs and subadults likely suggests that competition arises with an increasing number of younger individuals. A large number of cubs is often found in large African lion prides, which often have higher cub recruitment (Kissui and Packer 2004). Although these large groups benefit from increased prey capture success rates (Funston et al. 1998), per capita food intake is still lowered when prey are distributed among group members. This reduced cub per capita intake, which results from an increased number of young individuals, suggests that cub survival serves a key role in shaping female lion social organization in prides. Pride female lions exhibit egalitarian reproduction, producing equal numbers of cubs to foster cooperative investment in parental care while reducing reproductive skewness in prides (Packer et al. 2001). While egalitarian reproduction can promote group cohesion, increased competition for food during temporary food shortages may lead to prides splitting into subgroups. Thus, feeding competition becomes a key driver of fission–fusion dynamics in lions, an adaptive strategy to mitigate short‐term competition while maintaining the long‐term profits of group living (Mbizah et al. 2019).

The reduction in per capita food intake for subadults with increased numbers of each of the three demographic groups indicates that competition increases as the young lions mature. This competition may be important in the formation of smaller prides or the departure of grown‐up lions from prides, especially males. Increasing the number of individuals feeding on a carcass reduces individual consumption, which may force individuals to switch to being solitary or to form smaller groups (Ranta et al. 1993). The increased competition between subadults and adult female lions likely explains further reasons why mature male lions depart from their natal prides (Pusey and Packer 1987). Although previous work on African lions show that subadults may leave their natal groups to seek mating opportunities (Packer and Pusey 1997), our findings suggest that increased feeding competition in the natal groups could also drive males away. The time to leave natal prides seems like it is equivalent to “giving up time” in optimal foraging (Charnov 1976; McNair 1982; Pyke 1978). Numerous aspects of foraging are reported to affect offspring dispersal from natal groups in many species, including resource shortage, social facilitation, conspecific aggression, and developmental and environmental triggers (Clobert et al. 2009; Holekamp 1986; Missakian 1972; Stoinski et al. 2009; Yeager 1990). The advantage that subadults obtain when coexisting with both cubs and adult females suggest that subadults take advantage of feeding with the hunting group of the pride. In most large mammals, mature individuals show high prey capture success (MacNulty et al. 2009). Subadults may also benefit from social kin‐selected tolerance associated with kinship dynamics, which is common in African lions (Packer et al. 1990).

A two‐way interaction model best explained the effect of feeding group demographic structure on the per capita food intake of adult females, indicating that intake was influenced by both single demographic groups and the combination of different demographic groups. An increase in the size of African lion feeding groups of all age and sex categories was associated with decreased per capita intake for females. This indicates that female lions in a group did not gain foraging advantages, as Packer et al. (1990), suggesting that feeding cannot fully explain why lions form groups. The negative effects of cubs and subadults on adult female lion per capita food intake represent the tradeoffs between resource sharing and the demand to meet higher growth needs of young individuals. At younger ages, most mammals exhibit high food consumption rates to meet growth requirements (Archer et al. 1998; Bell et al. 2012; Huang et al. 2018; Laurenson 1995). Prioritizing food allocation to offspring is a common pattern in most social carnivores (Jordan et al. 2022). Although our findings are based on standardized assumptions of intake based on consumption rates of each demographic group, they reflect a group organization and feeding interactions between adult lions and their offspring.

Females may also tolerate younger individuals when feeding by collaborating with them, which may aid their learning and subsequent survival and contribution to adult female fitness. High per capita food intake obtained by adult females in rearing groups with many adults indicates the varied patterns in group foraging dynamics. Lions living in a pride likely gain both, increased fitness, through increased offspring survival, and increased energy intake. The additive effect of the number of adult males and females on the adult male per capita intake rate reflects that adult lions in prides exhibit competitive interactions in feeding. This may also indicate male dominance over other demographic groups in feeding. Dominance hierarchies in feeding are a common phenomenon in social predators like African lions and have been reported for Asiatic lions, too (Chakrabarti and Jhala 2017), and spotted hyena (Jarvey et al. 2022; Tilson and Hamilton 1984).

We acknowledge that our study has a number of limitations concerning the calculation of per capita food intake, which may introduce uncertainty. First, we used standardized biomass consumption rates of lions of different ages and sexes determined from common published values. Although sufficient to achieve our goal of assessing the direction of effects of feeding group demographic composition and prey body mass on food intake, rather than a precise estimation of intake by all individuals, we suggest that where animals are free to exhibit natural dynamic behaviors shaped by environmental factors (including those being tested), standardized values can be supplemented by field observation data. Information such as the number of feeding groups and age/sex composition is assumed accurate because observed lions are in our long‐term monitoring study in which all lions have been identified based on whisker masks, age, and sex. Second, prey body masses of all species (Pacifici et al. 2013) did not capture individual variability based on environmental and individual factors. Despite these limitations, our approach, which standardized our analyses by allowing multiple observations in our long‐term study across groups with varied compositions, number of individuals, and prey sizes, has reasonably balanced practicality and accuracy in the calculation of per capita intake.

## 5 Conclusions

Our analyses suggest that per capita food intake of individual lions depends on prey size and interactions among pride members at carcasses with a balance between competition and cooperation. Variations in estimated food intake in feeding groups with different demographic compositions imply that hierarchical, competitive, and facilitative mechanisms operate in prides. For example, lowered food intakes among young individuals and among adults suggest competitive interactions, while increased intakes in subadults and adult females in rearing groups reflect benefits of cooperative foraging. Moreover, our findings also suggest that although an increase in group size reduces intake, African lion group foraging exhibits cooperative dynamics that demonstrate high returns in food intake. While our study provides valuable information on the effect that demographic group composition of African lion prides has on per capita food intake, a few limitations arose and give a direction for future studies. In our study, we estimated the per capita food intakes based on reported consumption rates of each age‐sex class and estimated prey species masses. Further studies involving the actual per capita food intakes in different demographic structures in relation to individual traits and prey characteristics will shed more light on the African lion feeding dynamics.

## Author Contributions

**Thobias Oddo Tomeka:** conceptualization (equal), data curation (lead), formal analysis (lead), methodology (equal), resources (equal), software (equal), visualization (equal), writing – original draft (lead), writing – review and editing (equal). **Bernard M. Kissui:** conceptualization (equal), funding acquisition (lead), investigation (equal), methodology (equal), project administration (equal), resources (equal), supervision (supporting), writing – review and editing (equal). **Ifura Godfrey Ukio:** data curation (equal), investigation (equal), writing – review and editing (equal). **Frank R. Mushi:** data curation (supporting), investigation (equal), project administration (supporting), writing – review and editing (equal). **Rudolf F. Mremi:** conceptualization (equal), data curation (equal), formal analysis (supporting), writing – review and editing (equal). **Nathan J. Roberts:** conceptualization (equal), methodology (equal), writing – review and editing (equal). **Marcel Holyoak:** conceptualization (equal), formal analysis (supporting), methodology (equal), writing – review and editing (equal). **Guangshun Jiang:** conceptualization (equal), formal analysis (supporting), methodology (equal), resources (equal), supervision (lead), writing – review and editing (equal).

## Conflicts of Interest

The authors declare no conflicts of interest.

## Supporting information


Data S1.

## Data Availability

The data and code supporting the findings of this study are openly available in the Figshare repository at dx.doi.org/10.6084/m9.figshare.28435304 under CC BY 4.0 license.
