# Supplementary material for: Wild Ungulate Prey Size and Feeding Group Demographic Structure Affect Interactions and Per Capita Food Intake of African Pride Lions in East African Maasai Steppe
Source: Ecol Evol. 2025 Jul 28;15(8):e71787. doi: 10.1002/ece3.71787 (PMC12304429; doi:10.1002/ece3.71787)
Supplement: Supplementary file 1 — Data S1. [file ECE3-15-e71787-s001.docx]

Appendix Table S1 Model parameter description.

| **Variables** | **Description** |
| --- | --- |
| **Response variables** | |
| ${Per capita}_{\mathrm{cubs}}$ | Estimated amount of edible biomass available for each cub in a carcass |
| ${Per capita}_{\mathrm{Subadults}}$ | Estimated amount of edible biomass available for each subadult in a carcass |
| ${{Per capita}_{\mathrm{Adult}}}_{♀}$ | Estimated amount of edible biomass available for each adult female in a carcass |
| ${{Per capita}_{\mathrm{Adult}}}_{♂}$ | Estimated amount of edible biomass available for each adult male in a carcass |
| **Predictor variables** | |
| $N_{\mathrm{cubs}}$ | Number of cubs feeding on the carcass |
| $N_{\mathrm{subadults}}$ | Number of subadults feeding on a carcass |
| ${N_{\mathrm{Adult}}}_{♀}$ | Number of Adult females feeding on a carcass |
| ${N_{\mathrm{Adult}}}_{♂}$ | Number of adult males feeding on a carcass |

Appendix Table S2 Generalized Additive models for predicting the effect of group structure on carcass biomass per capita intake.

| **Model** | **Model formula** |
| --- | --- |
| **Single-predictor models** | |
| Model 1 | $\mathrm{Per}\mathrm{Capita}_{(demographic group)} \sim N_{\mathrm{cubs}}$ |
| Model 2 | $\mathrm{Per}\mathrm{Capita}_{(demographic group)} \sim N_{\mathrm{subadults}}$ |
| Model 3 | $\mathrm{Per}\mathrm{Capita}_{(demographic group)} \sim{N_{\mathrm{Adult}}}_{♀}$ |
| Model 4 | $\mathrm{Per}\mathrm{Capita}_{(demographic group)} \sim{N_{\mathrm{Adult}}}_{♂}$ |
| **Two-predictor models** | |
| Model 5 | $\mathrm{Per}\mathrm{Capita}_{(demographic group)} \sim N_{\mathrm{cubs}}+N_{\mathrm{subadults}}$ |
| Model 6 | $\mathrm{Per}\mathrm{Capita}_{(demographic group)} \sim N_{\mathrm{cubs}}+{N_{\mathrm{Adult}}}_{♀}$ |
| Model 7 | $\mathrm{Per}\mathrm{Capita}_{(demographic group)} \sim N_{\mathrm{cubs}}+{N_{\mathrm{Adult}}}_{♂}$ |
| Model 8 | $\mathrm{Per}\mathrm{Capita}_{(demographic group)} \sim N_{\mathrm{subadults}}+{N_{\mathrm{Adult}}}_{♀}$ |
| Model 9 | $\mathrm{Per}\mathrm{Capita}_{(demographic group)} \sim N_{\mathrm{subadults}}+{N_{\mathrm{Adult}}}_{♂}$ |
| Model 10 | $\mathrm{Per}\mathrm{Capita}_{(demographic group)} \sim{N_{\mathrm{Adult}}}_{♀}+{N_{\mathrm{Adult}}}_{♂}$ |
| **Three-predictor models** | |
| Model 11 | $\mathrm{Per}\mathrm{Capita}_{(demographic group)} \sim N_{\mathrm{cubs}}+N_{\mathrm{subadults}}+{N_{\mathrm{Adult}}}_{♀}$ |
| Model 12 | $\mathrm{Per}\mathrm{Capita}_{(demographic group)} \sim N_{\mathrm{cubs}}+N_{\mathrm{subadults}}+{N_{\mathrm{Adult}}}_{♂}$ |
| Model 13 | $\mathrm{Per}\mathrm{Capita}_{(demographic group)} \sim N_{\mathrm{cubs}}+{N_{\mathrm{Adult}}}_{♀}+{N_{\mathrm{Adult}}}_{♂}$ |
| Model 14 | $\mathrm{Per}\mathrm{Capita}_{(demographic group)} \sim N_{\mathrm{subadults}}+{N_{\mathrm{Adult}}}_{♀}+{N_{\mathrm{Adult}}}_{♂}$ |
| **Additive full model** (All four predictors) | |
| Model 15 | $\mathrm{Per}\mathrm{Capita}_{(demographic group)} \sim{N_{\mathrm{cubs}}+N}_{\mathrm{subadults}}+{N_{\mathrm{Adult}}}_{♀}+{N_{\mathrm{Adult}}}_{♂}$ |
| **Two-way interaction model** | |
| Model 16 | $\mathrm{Per}\mathrm{Capita}_{(demographic group)}\sim(N_{\mathrm{cubs}}+N_{\mathrm{subadults}}+{N_{\mathrm{Adult}}}_{♂}+{N_{\mathrm{Adult}}}_{♀})$2 |

Appendix Table S3 Generalized Cross-Validation (GCV) comparison table for a priori models predicting the effect of feeding group demographic structure on lion per capita intake for each age/sex category.

| **Model** | **GCV_Cubs_** | **GCV_Subadults_** | **GCV_Adult females_** | **GCV_Adult males_** |
| --- | --- | --- | --- | --- |
| **Single-predictor models** | | | | |
| **Model 1** | 5.598 | 15.992 | 22.997 | 23.028 |
| **Model 2** | 5.877 | 16.204 | 23.493 | 23.930 |
| **Model 3** | 5.980 | 16.440 | 24.668 | 24.895 |
| **Model 4** | 5.933 | 14.048 | 22.094 | 20.979 |
| **Additive two-predictor models** | | | | |
| **Model 5** | 5.504 | 15.359 | 20.760 | 22.609 |
| **Model 6** | 5.675 | 14.259 | 21.429 | 21.107 |
| **Model 7** | 5.669 | 16.187 | 22.836 | 23.330 |
| **Model 8** | 5.922 | 13.828 | 21.119 | 21.383 |
| **Model 9** | 5.929 | 16.214 | 22.942 | 24.322 |
| **Model 10** | 5.994 | 14.268 | 22.071 | 20.838 |
| **Additive three-predictor models** | | | | |
| **Model 11** | 5.5597 | 13.953 | 19.944 | 21.449 |
| **Model 12** | 5.5725 | 15.531 | 20.463 | 22.734 |
| **Model 13** | 5.7476 | 14.485 | 21.466 | 20.936 |
| **Model 14** | 5.980 | 14.043 | 20.979 | 21.231 |
| **Additive full model** | | | | |
| **Model 15** | 5.629 | 14.175 | 19.857 | 21.249 |
| **Two-way interaction model** | | | | |
| **Model 16** | 5.5721 | 13.673 | 18.749 | 21.001 |
